# RETRACTION: Beyond the Burn: An Observational Study of Cardiovascular Risk in Burn Survivors in the North of Iran

**DOI:** 10.1111/iwj.70661

**Published:** 2025-04-23

## Abstract

**RETRACTION:** SamadiS. , SalariA. , MobayenM. , ShakibaM. , BaziA. , HojjatiH. , PourN. H. , FarhadiB. , OtaghvarH. A. , ShirzadiA. , and FarzinM. , “Beyond the Burn: An Observational Study of Cardiovascular Risk in Burn Survivors in the North of Iran,” International Wound Journal 21, no. 3 (2024): e14794, 10.1111/iwj.14794.38420751 PMC10902762

The above article, published online on 29 February 2024 in Wiley Online Library (http://onlinelibrary.wiley.com/), has been retracted by agreement between the journal Editor in Chief, Professor Keith Harding; and John Wiley & Sons Ltd. A third party reported evidence of excessive self‐citations by the authors. The publisher did not find that self‐citations were unreasonable, given the topic. However, the publisher determined that the Methods were insufficiently supported by the references. It was also determined that there were significant problems with the methodology underlying the study, a finding which was supported by a Letter to the Editor published by the journal [1] which identified numerous flaws including the lack of a control group of non‐burn patients. The investigation also found that the ethics statement contradicts the informed consent procedure outlined in the Methods section. In view of the major errors and inconsistencies identified, the editors have decided to retract the article. The authors did not respond to our notice regarding the retraction.

Reference

[1] UmarM. , ChattopadhyayK. , ShamimL. , and VisariaA. , “Letter to Editor: Beyond the Burn: An Observational Study of Cardiovascular Risk in Burn Survivors in the North of Iran,” International Wound Journal 21, no. 6 (2024): e14794, 10.1111/iwj.14794.38899772 PMC11187797

**RETRACTION:** S. Samadi , A. Salari , M. Mobayen , M. Shakiba , A. Bazi , H. Hojjati , N. H. Pour , B. Farhadi , H. A. Otaghvar , A. Shirzadi , and M. Farzin , “Beyond the Burn: An Observational Study of Cardiovascular Risk in Burn Survivors in the North of Iran,” International Wound Journal 21, no. 3 (2024): e14794, 10.1111/iwj.14794.38420751 PMC10902762

The above article, published online on 29 February 2024 in Wiley Online Library (http://onlinelibrary.wiley.com/), has been retracted by agreement between the journal Editor in Chief, Professor Keith Harding; and John Wiley & Sons Ltd. A third party reported evidence of excessive self‐citations by the authors. The publisher did not find that self‐citations were unreasonable, given the topic. However, the publisher determined that the Methods were insufficiently supported by the references. It was also determined that there were significant problems with the methodology underlying the study, a finding which was supported by a Letter to the Editor published by the journal [1] which identified numerous flaws including the lack of a control group of non‐burn patients. The investigation also found that the ethics statement contradicts the informed consent procedure outlined in the Methods section. In view of the major errors and inconsistencies identified, the editors have decided to retract the article. The authors did not respond to our notice regarding the retraction.

**Reference**

[1] M. Umar , K. Chattopadhyay , L. Shamim , and A. Visaria , “Letter to Editor: Beyond the Burn: An Observational Study of Cardiovascular Risk in Burn Survivors in the North of Iran,” International Wound Journal 21, no. 6 (2024): e14794, 10.1111/iwj.14794.38899772 PMC11187797

